# The complete chloroplast genome of *Prunus domestica* L. (Rosaceae) and its phylogenetic implication

**DOI:** 10.1080/23802359.2020.1768928

**Published:** 2020-07-14

**Authors:** Wenjuan Geng, Liting Ouyang, Min Xu, Jianyong Jing

**Affiliations:** a College of Horticulture and Forestry, Xinjiang Agricultural University, Urumqi, China; b Laboratory of Germplasm Resources and Efficient Production of Horticultural Crops in Xinjiang, Urumqi, China

**Keywords:** *Prunus domestica*, complete chloroplast genome, phylogenetic analysis, *Rosaceae*

## Abstract

*Prunus domestica* commonly known as European plum is one of the most important wild fruit tree resources, and up to date, its wild community has only been found to be distributed in wild fruit forest area of the Tianshan Mountainsin Ili region of Xinjiang, China. Despite its agricultural importance and long history of cultivation, many questions remain about the origin of this species, due to absence of genome data. In this study, the complete chloroplast (cp) genome sequence of *P. domestica* was determined using next-generation sequencing. The entire cp genome was determined to be 157,395 bp in length. It contained large single-copy (LSC) and small single-copy (SSC) regions of 85,744 and 18,949 bp, respectively, which were separated by a pair of 26,351 bp inverted repeat (IR) regions. The genome contained 130 genes, including 85 protein-coding genes, 37 tRNA genes, and eight rRNA genes. The overall GC content of the genome is 36.8%. A phylogenetic tree reconstructed by 39 chloroplast genomes reveals that *P. domestica* is most closely related to *Prunus salicina*.

*Prunus L. s.l.* containing six subgenera, is mainly distributed in the northern hemisphere, including *P. domestica,* one of the most important wild fruit tree resources (Chase et al. 2016; Zhang et al. 2017). Up to date, its wild community has only been found to be distributed in wild fruit forest area of the Tianshan Mountainsin Ili region of Xinjiang, China (Lin and Shi 1989; Zhang et al. 1998). Despite its agricultural importance and long history of cultivation, many questions remain about the origin of this species, due to absence of genome data. So, it is necessary to develop genomic resources for *P. domestica* to provide basic intragenic information for the further study on phylogeny and breeding for genus *Prunus.*

Healthy leaf samples were collected from a wild *P. domestica* plant (43.38889 N,83.59198E, altitude 1235 m). The total genomic DNA was extracted from the fresh leaves of *P. domestica* using the DNeasy Plant Mini Kit (Qiagen, Valencia, CA, USA). The voucher specimen was deposited at Xinjiang agricultural university characteristic fruit tree research center (19sd-c-022). The whole genome sequencing was conducted on the Illumina Hiseq 4000 Sequencing System (Illumina, Hayward, CA). The filtered sequences were assembled using the program SPAdes assembler 3.10.0 (Bankevich et al. 2012). Annotation was performed using the DOGMA (Wyman et al. 2004). and tRNAscan (Schattner et al. 2005).

The plastome of *P. domestica* was determined to comprise double stranded, circular DNA of 157,395 bp containing two inverted repeat (IR) regions of 26,351 bp each, separated by large single-copy (LSC) and small single-copy (SSC) regions of 85,744 and 18,949 bp, respectively (Genome Warehouse acc. no. GWHALOD01000000; GenBank acc. no. MT302569). The genome contained 130 genes, including 85 protein-coding genes, 37 tRNA genes, and eight rRNA genes. The six protein-coding genes, six tRNA genes and four rRNA genes were duplicated in IR region. nineteen genes contained two exons and four genes (clpP and ycf3 and two rps12) contained three exons. The overall GC content of *P. domestica* cp genome is 36.8% and the corresponding values in LSC, SSC and IR regions are 34.6, 30.5 and 42.6%, respectively.

To investigate its taxonomic status, a maximum likelihood (ML) was reconstructed based on whole chloroplast genomes from 37 *Prunus* plants and two outgroup plant (*Neillia gracilis* and *Neillia serratisepala*) by Mafft version 1.4 and FastTree version 2.1.10 (Price et al. 2010). The ML phylogenetic tree shows that *P. domestica* is most closely related to *Prunus salicina*, with bootstrap support values of 100%. Chloroplast genomes of *P. domestica* adds valuable information for understanding the phylogenetic position of *P. domestica* in the subgenera *Prunus*. And more genome data of *Prunus* are needed to reveal the origin of *P. domestica* (Figure 1).

**Figure 1.**
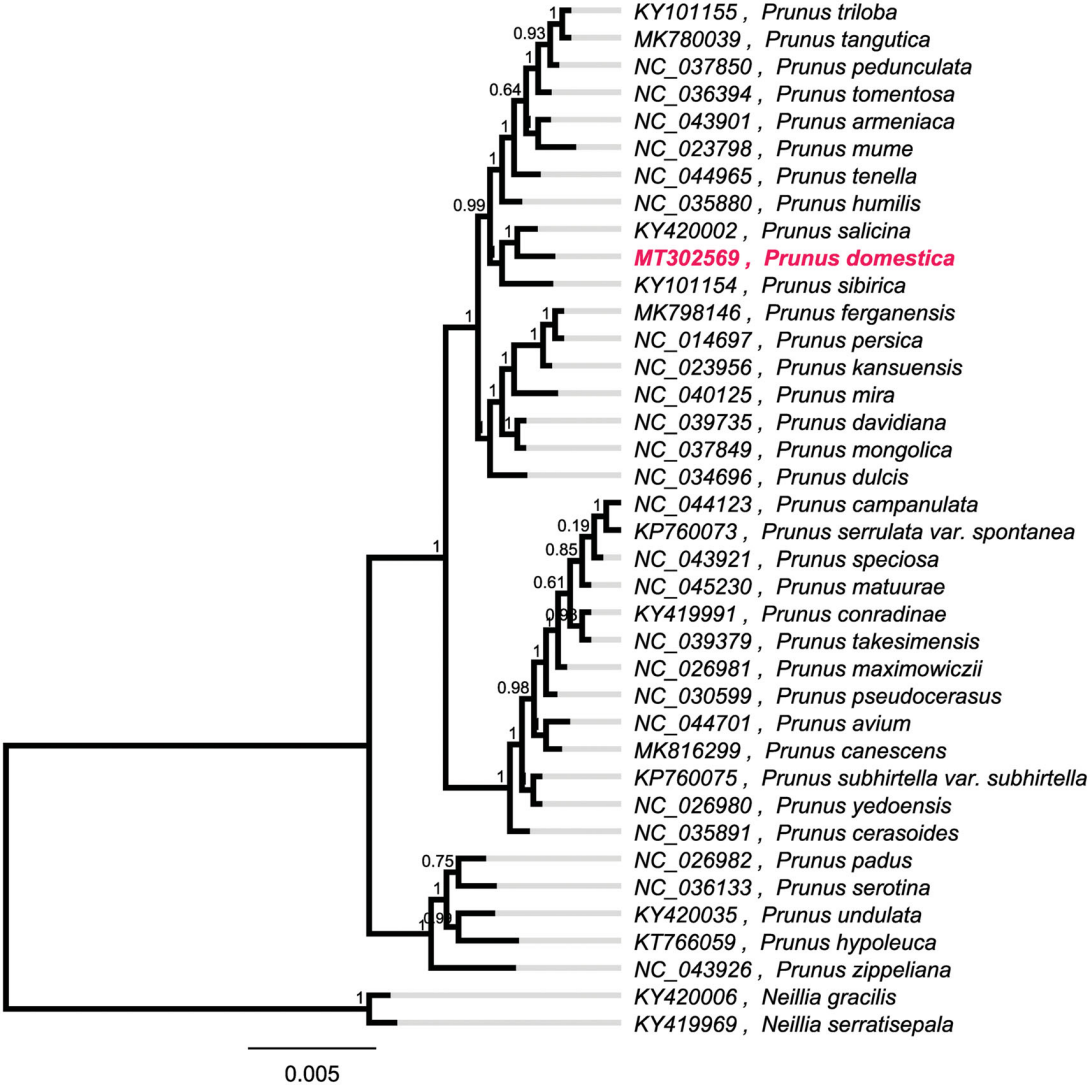
Maximum-likelihood phylogenetic tree based on whole chloroplast genomes from 37 *Prunus* plants and two outgroup plant (*Neillia gracilis* and *Neillia serratisepala*) and the support values are shown at the branches.

## Data Availability

The complete chloroplast genome sequence of *Prunus domestica* is deposited in the Genome Warehouse (https://bigd.big.ac.cn/search?dbId=gwh&q=GWHALOD01000000&page=1).
